# TSPO PET signal using [^18^F]GE180 is associated with survival in recurrent gliomas

**DOI:** 10.1007/s00259-022-06006-1

**Published:** 2022-11-04

**Authors:** Stefanie Quach, Adrien Holzgreve, Lena Kaiser, Marcus Unterrainer, Franziska J. Dekorsy, Debie V. Nelwan, Laura M. Bartos, Sabrina V. Kirchleitner, Jonathan Weller, Lorraine Weidner, Maximilian Niyazi, Viktoria C. Ruf, Jochen Herms, Sophia Stöcklein, Christian Wetzel, Markus J. Riemenschneider, Louisa v. Baumgarten, Niklas Thon, Matthias Brendel, Rainer Rupprecht, Peter Bartenstein, Joerg-Christian Tonn, Nathalie L. Albert

**Affiliations:** 1 Department of Neurosurgery, University Hospital, LMU Munich, Marchioninistr. 15, 81377 Munich, Germany; 2 Department of Nuclear Medicine, University Hospital, LMU Munich, Marchioninistr. 15, 81377 Munich, Germany; 3 Department of Radiology, University Hospital, LMU Munich, Munich, Germany; 4 Department of Neuropathology, Regensburg University Hospital, Regensburg, Germany; 5 Department of Radiation Oncology, University Hospital, LMU Munich, Munich, Germany; 6 Present Address: German Cancer Consortium (DKTK), Partner Site Munich, German Cancer Research Center (DKFZ), Heidelberg, Germany; 7 Center for Neuropathology and Prion Research, LMU Munich, Munich, Germany; 8 Department of Psychiatry and Psychotherapy, Molecular Neurosciences, University of Regensburg, Regensburg, Germany

**Keywords:** TSPO, [^18^F]GE180 PET, Glioma, Survival, Prognostic biomarker

## Abstract

**Purpose:**

Glioma patients, especially recurrent glioma, suffer from a poor prognosis. While advances to classify glioma on a molecular level improved prognostication at initial diagnosis, markers to prognosticate survival in the recurrent situation are still needed. As 18 kDa translocator protein (TSPO) was previously reported to be associated with aggressive histopathological glioma features, we correlated the TSPO positron emission tomography (PET) signal using [^18^F]GE180 in a large cohort of recurrent glioma patients with their clinical outcome.

**Methods:**

In patients with [^18^F]GE180 PET at glioma recurrence, [^18^F]GE180 PET parameters (e.g., SUV_max_) as well as other imaging features (e.g., MRI volume, [^18^F]FET PET parameters when available) were evaluated together with patient characteristics (age, sex, Karnofsky-Performance score) and neuropathological features (e.g. WHO 2021 grade, *IDH*-mutation status). Uni- and multivariate Cox regression and Kaplan–Meier survival analyses were performed to identify prognostic factors for post-recurrence survival (PRS) and time to treatment failure (TTF).

**Results:**

Eighty-eight consecutive patients were evaluated. TSPO tracer uptake correlated with tumor grade at recurrence (*p* < 0.05), with no significant differences in *IDH*-wild-type versus *IDH*-mutant tumors. Within the subgroup of *IDH*-mutant glioma (*n* = 46), patients with low SUV_max_ (median split, ≤ 1.60) had a significantly longer PRS (median 41.6 vs. 25.3 months, *p* = 0.031) and TTF (32.2 vs 8.7 months, *p* = 0.001). Also among *IDH*-wild-type glioblastoma (*n* = 42), patients with low SUV_max_ (≤ 1.89) had a significantly longer PRS (median not reached vs 8.2 months, *p* = 0.002). SUV_max_ remained an independent prognostic factor for PRS in the multivariate analysis including CNS WHO 2021 grade, *IDH* status, and age. Tumor volume defined by [^18^F]FET PET or contrast-enhanced MRI correlated weakly with TSPO tracer uptake. Treatment regimen did not differ among the median split subgroups.

**Conclusion:**

Our data suggest that TSPO PET using [^18^F]GE180 can help to prognosticate recurrent glioma patients even among homogeneous molecular subgroups and may therefore serve as valuable non-invasive biomarker for individualized patient management.

**Graphical abstract:**

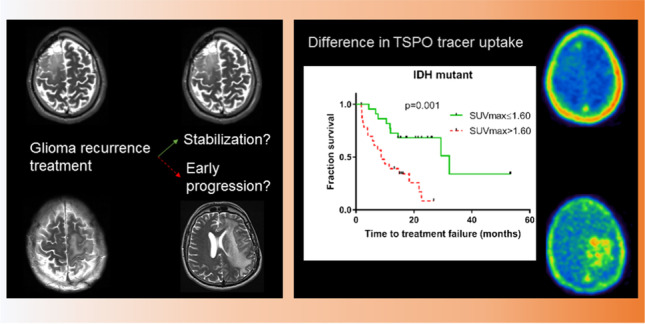

**Supplementary Information:**

The online version contains supplementary material available at 10.1007/s00259-022-06006-1.

## Introduction

Diffuse gliomas are the most frequent adult primary brain tumors [1], and almost all relapse after initial treatment. Efforts to understand the molecular mechanisms and prognostic factors of these heterogeneous tumors have led to the discovery of molecular markers which shape the most recent 2021 World Health Organization (WHO) classification of brain tumors to distinguish different glioma subgroups [2]. This molecular classification is mirrored in the current treatment guidelines [3]. All guidelines emphasize magnetic resonance imaging (MRI) as the gold standard for diagnostic imaging to gain information about the presumable histology and composition of the lesion as well its extent and, as a consequence, its amenability for treatment and its potential prognosis [4]. Beyond MRI, amino acid PET such as [^18^F]Fluoroethyltyrosine (FET) positron emission tomography (PET) has proven valuable to delineate tumor extent, identify intratumoral heterogeneity, and distinguish recurrent disease from pseudoprogression [5–7]. Subsequently, amino acid PET has entered current diagnostic guidelines for primary and recurrent glioma [8, 9]. However, it is becoming increasingly clear that the interplay between tumor cells and the tumor microenvironment plays an important role in disease progression and treatment response or resistance. In this context, tumor-associated macrophages and microglia gain considerable attention, also in recurrent glioma [10–12]. Thus, PET imaging of the respective cellular elements is of interest to provide insight into the tumor microenvironment and tumor-host interaction. As such, PET imaging of the 18 kDa translocator protein (TSPO) as a marker of activated microglia and neuroinflammation [13] has shown increased uptake in glioma patients, as well [14–17].

TSPO is a mitochondrial membrane protein with a variety of functions in health and disease. Beyond classical mitochondrial functions such as respiration and oxidative stress regulation, more diverse functions such as cell proliferation and apoptosis have recently been implied [18]. TSPO is expressed ubiquitously and upregulated in steroid-synthesizing cells and microglial and malignant cells [18].

Preliminary data show an upregulation of TSPO expression in high-grade glioma and hint at a correlation between histologically increased TSPO expression and shorter survival, yet this was before description of molecularly defined glioma subgroups [19, 20]. To visualize TSPO expression and its spatial distribution in vivo, different radiolabeled TSPO ligands such as [^11^C]-(R)PK11195 were used and shown to correlate with histological TSPO expression [21], but usability was limited by a low binding affinity or a short half-life of [^11^C]. In contrast, the third-generation TSPO radioligand [^18^F]GE180 shows a high binding affinity [22] and convenient half-life for the clinical use due to the labelling with [^18^F]. In glioma patients, tracer uptake volumes were reported to exceed areas of contrast enhancement on MRI [23]. Several clinical case series showed a trend of higher TSPO tracer uptake in histologically or molecular biologically more aggressive tumors, such as isocitrate dehydrogenase (*IDH*) wild-type tumors [24, 25]. We here aim to describe the relationship between TSPO tracer uptake and clinical outcome in molecularly defined groups of recurrent glioma patients.

## Methods

### Patients

Eligible were patients with a histologically verified glioma and an [^18^F]GE180 PET at recurrence as suspected by Response Assessment in Neuro-Oncology (RANO) criteria [26] between 2016 and 2020. All patients with histologically or clinically verified progression were included in the analysis. All patients provided written informed consent. The study was approved by the local ethics committee (No. 16–601, 17–769).

### Imaging acquisition and analysis

All PET scans were performed on a Biograph 64 PET/CT scanner (Siemens, Erlangen, Germany). Tracer production and image acquisition were performed as described previously [25]. For [^18^F]GE180 PET, approximately 180 MBq [^18^F]GE180 were injected as an intravenous bolus, and summation images 60–80 min post injection (p. i.) were used for image analysis (Hermes Medical Solutions, Stockholm, Sweden). In order to exclude potential confounding factors related to treatment or inflammatory responses in the background, maximal tumor uptake was assessed as maximum standardized uptake value (SUV_max_) for [^18^F]GE180 PET analyses, whereas for FET PET, maximum tumor-to-background ratio (TBR_max_) was used as recommended in the joint EANM/EANO/RANO guideline [9].

For [^18^F]FET PET, approximately 180 MBq [^18^F]FET were injected and 20–40 min p.i. summation images were analyzed using a Hermes workstation (Hermes Medical Solutions, Stockholm, Sweden). Because of low inter- and intra-rater variability [27], the mean background activity was defined as the mean activity of at least 6 crescent-shaped cortical areas in the healthy contralateral side, and SUV_max_ was divided by the mean background activity to obtain TBR_max_. The biological tumor volume was semiautomatically delineated using the histologically verified [28, 29] and recommended [9] standard 1.6 background activity as threshold. Routine MRI included gadolinium-enhanced T1- and non-enhanced T1- and T2-weighted images. MRI volumes were calculated using BRAINLAB ELEMENTS™ (Brainlab AG, Munich, Germany).

### Neuropathological analysis

All tumors were classified according to WHO 2016 [30] at the Center for Neuropathology and Prion Research of the University of Munich and re-classified according to WHO 2021 [2] retrospectively for the purpose of this study. *IDH* mutation status was determined by pyrosequencing. Sanger sequencing was used to detect telomerase reverse transcriptase (*TERT*) promoter mutations, as well as microsatellite analysis for detection of 1p and 19q deletions. O^6^-methylguanine-DNA methyltransferase (*MGMT*) promoter methylation was analyzed by methylation-specific PCR and sequencing analysis. *MGMT* promoter methylation status was classified dichotomously methylated when 13 or more of 25 sites were methylated; otherwise, as unmethylated [31]. Genotyping for the genetic polymorphism of the TSPO gene was performed as previously described [23].

### Data analyses

Post-recurrence survival (PRS) was defined as the time between MRI suggestive of recurrence initiating PET imaging and date of death. Time to treatment failure after recurrence (TTF) was defined as the time between MRI showing recurrence and MRI showing further recurrence according to RANO criteria. Systemic post recurrence therapies were categorized as combined radio-and chemotherapy, radiotherapy only, chemotherapy only, experimental or others, and no tumor-specific therapy. Categorical variables were compared by *χ*^2^ test, and continuous variables were compared by the Mann–Whitney *U* test. Pearson’s correlation coefficient was used to test for correlation between two continuous variables. Uni- and multivariate Cox regression and Kaplan–Meier survival analyses were performed to identify prognostic factors for post-recurrence survival (PRS) and time to treatment failure (TTF), respectively. *p* < 0.05 was considered statistically significant. Statistical analyses were performed using SPSS statistics version 23 (IBM, Armonk, New York, USA).

## Results

### Patients

[^18^F]GE180 PET scans of 88 recurrent glioma patients were evaluated. Median age was 49 years (range 23.6–71.9). Fifty-six (63.6%) patients were male, 32 female (36.4%). Forty-two (47.7%) tumors were diagnosed as *IDH*-wild-type glioblastoma, 46 (52.3%) as *IDH*-mutant glioma. Among all 28 *IDH*-mutant astrocytoma, 10 were classified as WHO 2021 grade 4, 17 as WHO grade 3, and one as WHO grade 2, respectively. Eighteen tumors were classified as oligodendroglioma, *IDH*-mutant, and 1p/19q-codeleted; 11 WHO grade 3; and 7 WHO grade 2.

Median follow-up time was 15.6 months (95% confidence interval (CI): 13.3–18.0 months). In 59 (67.0%) cases, tumor recurrence was verified histologically; in all other cases, clinical deterioration confirmed tumor recurrence. All cases of low-grade tumors with new contrast enhancement or new [^18^F]FET enhancement were histologically verified to prove or rule out malignant transformation. For recurrence treatment, radiotherapy was performed in 60 cases (68.2%), 26 of them (29.5% of all patients) in combination with chemotherapy. Chemotherapy alone was administered in 22 cases (25.0%), 15 (17.0%) patients underwent craniotomy and tumor resection, 3 (3.4%) received other/experimental treatment, and 3 received best supportive care due to clinical deterioration or refusing therapy. TSPO binding affinity status was available for 78 patients; of these, 7 (9.0%) were low-affinity binders, 27 (34.6%) were medium-affinity binders, and 44 (56.4%) were high-affinity binders.

### PET specifications according to patient groups

SUV_max_ values were correlated with 2021 CNS WHO grade (Table 1). No significant difference was found between recurrent *IDH*-mutant and *IDH*-wild-type tumors (Table 1).

**Table 1:** [^18^F]GE180 uptake characteristics in the examined patient population

*All recurrent gliomas*	SUV_max_ (median (range))	*p*-value
*Overall (n* = *88)*	1.68 (0.59–4.36)	
*Male (n* = *56)* *Female (n* = *32)*	1.73 (0.59–3.83) 1.56 (0.59–4.36)	0.370
*CNS WHO 2021 grade 2 (n* = *8)* *CNS WHO 2021 grade 3 (n* = *28)* *CNS WHO 2021 grade 4 (n* = *52)*	0.90 (0.59–3.83) 1.45 (0.59–3.82) 1.91 (0.85–3.83)	0.031
*IDH-mutant (n* = *46)* *IDH-wild type (n* = *42)*	1.60 (0.59–4.36) 1.89 (0.85–3.83)	0.071
*IDH mut. − 1p/19 codel (n* = *28)* *IDH mut.* + *1p/19 codel (n* = *18)*	1.72 (.59–3.79) 1.28 (.61–4.36)	0.714
*IDH wt, TERT wildtype (n* = *5)* *IDH wt, TERT mutant (n* = *22)*	1.70 (1.29–3.83) 1.89 (1.00–3.12)	0.909
*IDH wt, MGMT methylated (n* = *19)* *IDH wt, MGMT unmethylated (n* = *22)*	2.29 (0.85–3.83) 1.81 (1.00–3.03)	0.114
*Low-affinity binding status (n* = *7)* *Medium-affinity binding status (n* = *27)* *High-affinity binding status (n* = *44)*	2.32 (0.80–3.79) 1.76 (0.59–3.08) 1.58 (0.61–3.83)	0.231

*SUV*_*max*_ maximum standardized uptake value, *CNS WHO 2021* World Health Organization Classification of Tumors of the Central Nervous System, *IDH* isocitrate dehydrogenase, *TERT* telomerase reverse transcriptase

### Post-recurrence treatment

Among all 42 patients with *IDH*-wild-type glioblastoma, 3 patients with a sub-median SUV_max_ and 2 patients with a supra-median SUV_max_ underwent craniotomy and tumor resection as part of their recurrence treatment (*p* = 0.89). Among patients with *IDH*-mutant tumors, 7 with a sub-median SUV_max_ and 3 with a supra-median SUV_max_ underwent craniotomy and tumor resection (*p* = 0.36). Systemic post-recurrence therapies did not differ between patients with a sub- or supra-median SUV_max_ (*p* = 0.22 for *IDH*-wild type, *p* = 0.30 for *IDH*-mutant tumors (Chi-square test, Table 2).

**Table 2:** Treatment regimens in patients with lower or higher than median SUV_max_

	SUV_max_ ≤ median (*n*; %)	SUV_max_ > median (*n*; %)	*p*-value
*IDH-wildtype (n* = *42)* *Radio-and chemotherapy* *Radiotherapy only* *Chemotherapy only* *Experimental/others* *No tumor-specific therapy*	21 (100) 7 (33.3) 7 (33.3) 4 (19.0) 0 (0.0) 3 (14.3)	21 (100) 5 (23.8) 8 (38.1) 6 (28.6) 2 (9.5) 0 (0.0)	0.215
*IDH-mutant (n* = *46)* *Radio-and chemotherapy* *Radiotherapy only* *Chemotherapy only* *Experimental/others*	23 (100) 7 (30.4) 12 (52.2) 4 (17.4) 0 (0.0)	23 (100) 7 (30.4) 7 (30.4) 8 (34.8) 1 (4.3)	0.302

*SUV*_*max*_ maximum standardized uptake value, *IDH* isocitrate dehydrogenase

### Post-recurrence survival and time to treatment failure

Overall, uptake intensity on [^18^F]GE180 PET at recurrence was highly associated with patients’ outcome: patients with low SUV_max_ (≤ 1.68; median split) survived more than three times longer than those with high SUV_max_ (median PRS 41.6 vs. 12.6 months; *p* < 0.01) (Table 3). Also, the TTF was significantly longer in cases with low SUV_max_ compared to high SUV_max_ (14.9 vs. 6.2 months; *p* < 0.01) (Table 3). Other significant factors in the univariate analysis were *IDH* mutation status and CNS WHO 2021 grade for TTF (both *p* < 0.01) and PRS (*p* < 0.01 and *p* < 0.01). In the multivariate analysis including CNS WHO 2021 grade, *IDH* status, and age, SUV_max_ remained an independent significant factor for PRS (*p* = 0.03) and TTF (*p* = 0.03), whereas CNS WHO 2021 grade (*p* = 0.02) was the only other independent factor for TTF. Accordingly, the association between uptake intensity on [^18^F]GE180 PET and outcome was likewise found in the subgroups of molecularly defined tumors: for patients with recurrent *IDH*-wild-type glioblastoma, median PRS after recurrence was 8.2 months for patients with an SUV_max_ higher than the median of 1.89, and not reached for patients with a sub-median SUV_max_ (*p* < 0.01). Median TTF after recurrence was 6.1 months for all IDH-wild-type glioblastoma (6.8 for sub-median vs. 5.4 months for supra-median SUV_max_, *p* = 0.14).

**Table 3:** Survival of recurrent glioma patient groups according to tracer uptake

*All recurrent glioma cases*	PRS (median; months)	*p*-value	TTF (median; months)	*p*-value
*All diagnoses (n* = *88)* *SUV*_*max*_ ≤ *1.68 (n* = *44)* *SUV*_*max*_ > *1.68 (n* = *44)*	27.9 41.6 12.6	< 0.001	8.7 14.9 6.2	< 0.001
*All IDH-wildtype (n* = *42)* *SUV*_*max*_ ≤ *1.89 (n* = *21)* *SUV*_*max*_ > *1.89 (n* = *21)*	10.6 Not reached 8.2	0.002	6.1 5.4 6.8	0.142
*All IDH-mutant (46)* *SUV*_*max*_ ≤ *1.60 (n* = *23)* *SUV*_*max*_ > *1.60 (n* = *23)*	36.9 41.6 25.3	0.031	18.4 32.2 8.7	0.001
*All astrocytoma, IDH-mutant (28)* *SUV*_*max*_ ≤ *1.72 (14)* *SUV*_*max*_ > *1.72 (14)*	27.9 36.9 13.5	0. 009	11.7 22.6 6.2	0.007
*Astrocytoma WHO 2021 grade 3, IDH mutant (17)* *SUV*_*max*_ ≤ *1.55 (9)* *SUV*_*max*_ > *1.55 (8)*	36.9 36.9 13.1	0.015	11.7 32.2 2.7	0.025
*All low-grade (2 or 3) astrocytoma, IDH-mutant (18)* *SUV*_*max*_ ≤ *1.33 (9)* *SUV*_*max*_ > *1.33 (9)*	36.9 36.9 13.1	0.003	14.5 32.2 6.2	0.063
*All oligodendroglioma, IDH-mutant and 1p/19q codeleted (18)* *SUV*_*max*_ ≤ *1.28 (9)* *SUV*_*max*_ > *1.28 (9)*	Not reached Not reached Not reached	0.464	Not reached Not reached 18.4	0.147

*SUV*_*max*_ maximum standardized uptake value, *IDH* isocitrate dehydrogenase, *PRS* post recurrence survival, *TTF* time to treatment failure

**Fig. 1:**
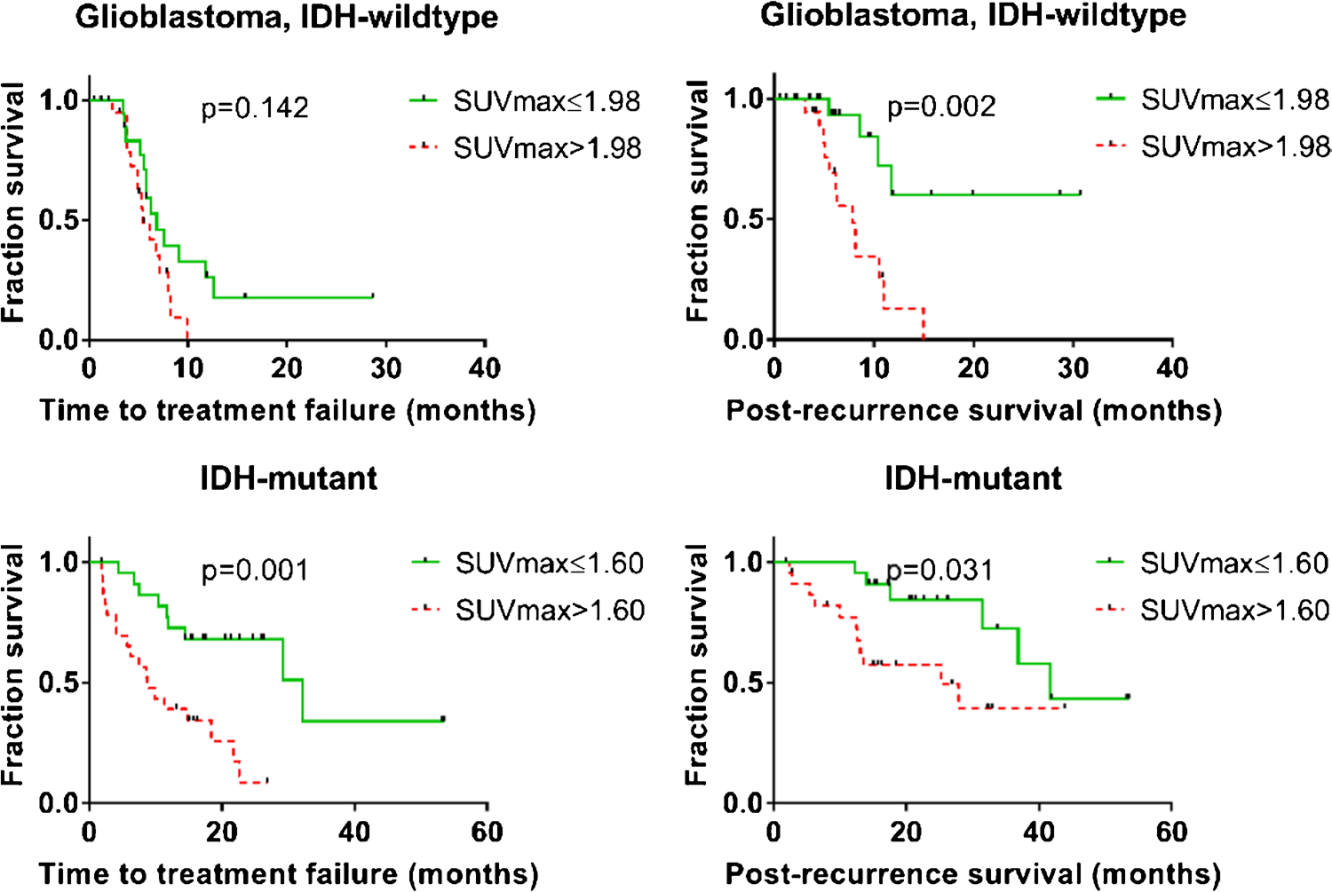
Time to treatment failure and post recurrence survival in IDH-wild-type and IDH-mutant glioma patients according to maximum [^18^F]GE180 uptake. SUV_max_ maximum standardized uptake value, IDH isocitrate dehydrogenase

**Fig. 2:**
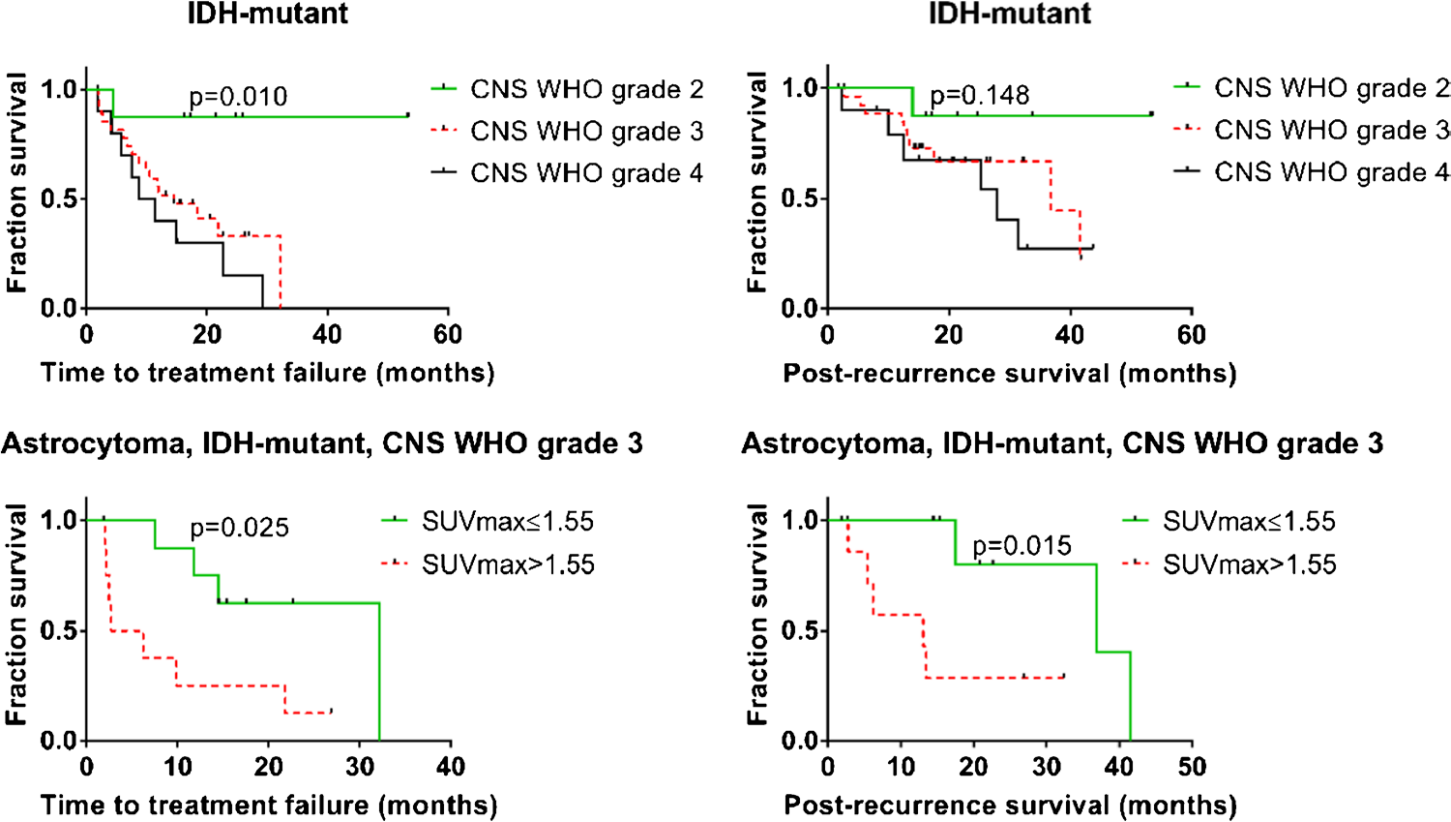
Survival of IDH-mutant astrocytoma. SUV_max_ maximum standardized uptake value, IDH isocitrate dehydrogenase

**Fig. 3:**
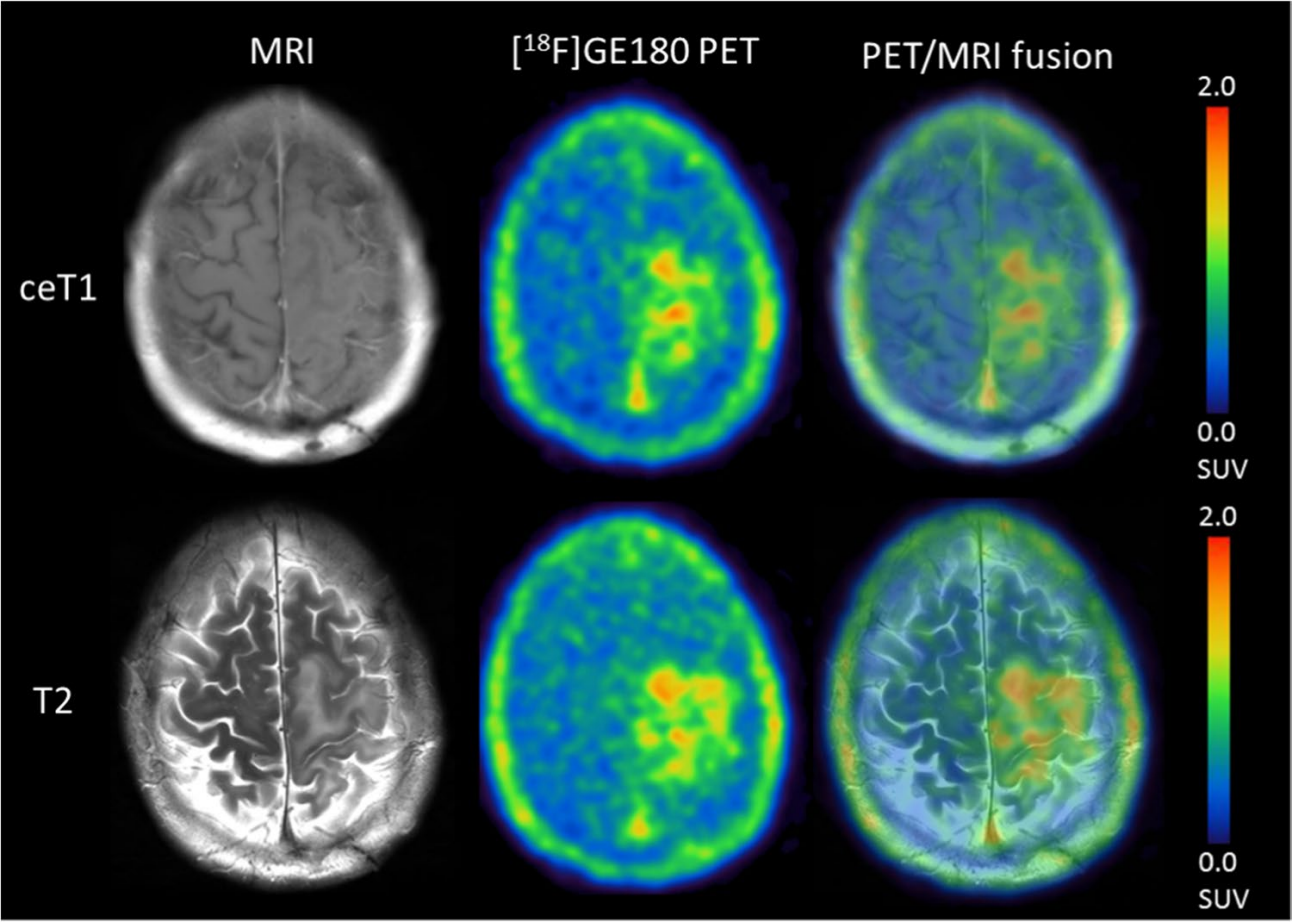
Exemplary case of a 39.5-year-old patient with recurrent astrocytoma, *IDH*-mutant, CNS WHO 2021 grade 3 (SUV_max_ 2.85). The patient progressed soon after re-radiochemotherapy and died 6 months after recurrence

**Fig. 4:**
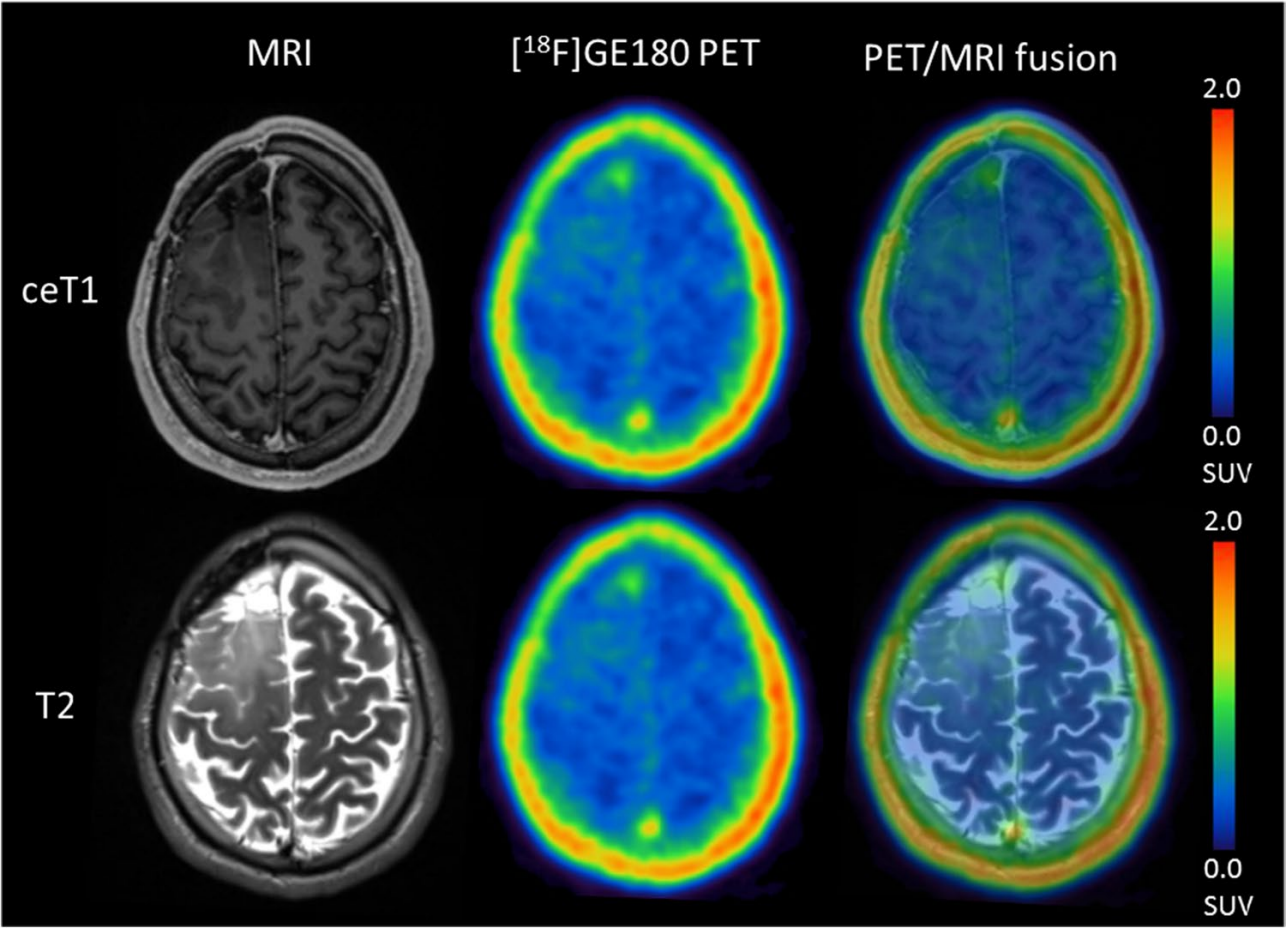
Exemplary case of a 31.0-year-old patient with histologically verified recurrent astrocytoma, *IDH*-mutant, CNS WHO 2021 grade 3 (SUV_max_ 0.79, Ki67 at recurrence 20%). The patient remained stable 15 months after recurrence and re-radiochemotherapy

Among patients with *IDH*-mutant tumors, median PRS was 36.9 months, and median TTF was 18.4 months (Table 3). PRS was significantly longer in patients with low SUV_max_ (≤ 1.60; median split; 41.6 vs. 25.3 months, *p* = 0.03, see Fig. 1). This difference was also found for median TTF (32.2 vs 8.7 months, *p* < 0.01), also in the subgroups of all astrocytoma, *IDH*-mutant (22.6 vs 6.2 months, *p* = 0.01 for TTF, 36.9 vs 13.5, *p* = 0.01 for PRS), and even within the very homogeneous subgroup of CNS WHO 2021 grade 3 astrocytoma, *IDH*-mutant (32.2 vs 2.7 months, *p* = 0.03 for TTF, 36.9 vs 13.1, *p* = 0.02 for PRS) (Table 3 and Fig. 2). Exemplary cases of *IDH-*mutant tumors with supra- or sub-median SUV_max_ are shown in Figs. 3 and 4. The small subgroup of oligodendroglioma, *IDH*-mutant and 1p/19q codeleted, did not have enough events for a separate statistical evaluation (Table 3).

### Subgroup analyses

Among patients with *IDH*-wild-type glioblastoma and a high [^18^F]GE180 SUV_max_, slightly more tumors (11/21) were *MGMT*-methylated than in the subgroup of patients with a sub-median SUV_max_ (8/20, Table 4). *MGMT-*methylated tumors tended to be treated with chemotherapy, either alone or combined with radiotherapy, more often than unmethylated tumors (63.2% vs 45.5%, *p* = 0.26). In the 35 *IDH*-wild-type glioblastoma for whom additional [^18^F]FET PET was available, [^18^F]FET TBR_max_ was higher in the subgroup with supra-median [^18^F]GE180 uptake (3.44 vs 3.10, *p* = 0.05), while [^18^F]FET PET-based volume did not differ significantly (Table 4). Inversely, no significant difference in TTF (sub-median 6.8 vs supra-median 7.2 months) or PRS (sub-median 10.4 vs supra-median 11.8 months, both p = 0.95) could be seen between patients with a TBR_max_ higher or lower than the median of 3.18 (Supplementary Fig. 1).

**Table 4:** Characteristics of high and low [^18^F]GE180 uptake groups

	SUV_max_ ≤ median (*n*; % or median; range)	SUV_max_ > median (*n*; % or median; range)	*p*-value
*IDH-wildtype (n* = *42)* * Male/female sex* * MGMT methylated/unmeth. (n* = *41)* * TERT wild type/mutant (n* = *27)* * Age: [years]* * [*^*18*^*F]FET TBR*_*max*_* (n* = *35)* * [*^*18*^*F]FET tumor volume: [ml] (n* = *35)* * T2 volume: [ml]* * Contrast volume: [ml]*	21 (50.0) 14/7 (33.3/16.7) 8/12 (19.5/29.3) 4/11 (14.8/40.7) 55.5 (32.3–70.0) 3.10 (1.55–4.83) 12.60 (0.0–76.66) 50.80 (0.0–198.10) 3.74 (.27–66.2)	21 (50.0) 15/6 (35.7/14.3) 11/10 (26.8/24.4) 1/11 (3.7/40.7) 55.8 (30.8–70.2) 3.44 (2.47–5.28) 28.71 (4.49–124.75) 80.10 (8.09–337.3) 16.50 (0.0–85.6)	0.739 0.427 0.223 0.858 0.045 0.109 0.075 0.022
*IDH-mutant (n* = *46)* * Male/female sex* * CNS WHO 2021 grade 2/3/4* * MGMT methylated/unmeth* * TERT wildtype/mutant (n* = *28)* * Age: [years]* * [*^*18*^*F]FET TBR*_*max*_* (n* = *42)* * [*^*18*^*F]FET tumor volume: [ml] (n* = *42)* * T2 volume: [ml]* * Contrast volume: [ml]*	23 (50.0) 12/11 (26.1/23.9) 7/15/1 (15.2/32.6/2.2) 20/3 (43.5/6.5) 9/5 (32.1/17.9) 47.5 (23.6–66.2) 2.51 (1.21–5.29) 5.14 (0.0–100.87) 50.7 (13.90–226.50) 0.06 (0.0–12.70)	23 (50.0) 15/8 (32.6/17.4) 1/13/9 (2.2/28.3/19.6) 18/5 (39.1/10.9) 10/4 (35.7/14.3) 39.8 (29.1–71.9) 4.15 (2.88–7.49) 33.08 (1.67–172.04) 59.15 (9.13–253.90) 10.10 (0.0–61.2)	0.369 0.004 0.437 0.686 0.456 < 0.001 0.002 0.303 0.002
*Astro, IDH mut., grade 3 (n* = *17)* * Male/female sex* * MGMT methylated/unmeth* * Age: [years]* * [*^*18*^*F]FET TBR*_*max*_ * [*^*18*^*F]FET tumor volume: [ml]* * T2 volume: [ml]* * Contrast volume: [ml]*	9 7/2 (41.2/11.8) 7/2 (41.2/11.8) 45.1 (29.9–56.3) 2.01 (1.56–3.82) 3.21 (0.00–71.96) 39.10 (15.70–226.50) 0.00 (0.00–12.20)	8 6/2 (35.3/11.8) 5/3 (29.4/17.6) 37.4 (30.4–57.6) 4.91 (3.08–5.86) 78.87 (11.44–172.04) 63.50 (12.40–110.10) 16.04 (3.84–61.20)	0.893 0.490 0.198 0.001 0.016 0.842 0.013

*SUV*_*max*_ maximum standardized uptake value, *IDH* isocitrate dehydrogenase, *TERT* telomerase reverse transcriptase, *[*^*18*^*F]FET* [^18^F]Fluoroethyltyrosine, *TBR*_*max*_ maximum tumor-to-brain-ratio, *CNS WHO 2021* World Health Organization Classification of Tumors of the Central Nervous System

In *IDH*-mutant glioma, tumors with a sub- or supra-median [^18^F]GE180-uptake showed a significantly different median [^18^F]FET volume (5.14 vs 33.08, *p* < 0.01), TBR_max_ (2.51 vs 4.15, *p* < 0.01) as well as contrast-enhancing volume on MRI (0.06 vs 10.10cm^3^, *p* < 0.01) (Table 4). Similar results were found in the subgroup of patients with grade 3 astrocytoma, *IDH*-mutant (Table 4). Patients with *IDH*-mutant glioma and an [^18^F]FET TBR_max_ higher than the median of 3.53 had a shorter median TTF (10.5 vs 29.3 months, *p* = 0.03), but not significantly different PRS compared to patients with a sub-median [^18^F]FET TBR_max_ (27.9 vs 41.6 months, *p* = 0.21) (Supplementary Fig. 1).

Among IDH-wild-type glioblastoma patients, 28 (66.7%) were scanned at the time of their first, 11 (26.2%) at their second, and 3 (7.1%) at their third recurrence. Among IDH-mutant glioma patients, 18 (39.1%) had their first, 14 (30.4%) their second, and 14 (30.4%) a third or later recurrence at the time of the scan. In the IDH-wild-type group, there was an intercorrelation between the number of recurrences and [^18^F]GE180 SUV_max_ with highest SUV_max_ values at latest recurrence. However, the recurrence stages did not result in different median PRS or TTF in this group (Supplementary table 1). In IDH-mutant tumor patients, contrariwise, a longer median TTF and PRS was found after their first or second recurrence, yet no significant correlation between recurrence stage and SUV_max_ was found here (Supplementary table 1).

Among all patients, there was a low to moderate correlation between [^18^F]GE180 SUV_max_ and both [^18^F]FET tumor volume (*r* = 0.490, *p* < 0.01), volume in contrast-enhanced MRI (*r* = 0.47, *p* < 0.01), and T2 MRI volume (*r* = 0.30, *p* < 0.01).

### Analyses according to [^18^F]GE180 TBR_max_

Among all patients, CNS WHO 2021 grade correlated with [^18^F]GE180 TBR_max_ (Supplementary table 2). Recurrence treatments did not differ between patients with a supra- or submedian TBR_max_ (Supplementary table 3). No significant difference in survival between supra- or sub-median TBR_max_ was observed among all patients or *IDH*-wild-type patients (Supplementary table 4). Among *IDH*-mutant and astrocytoma, *IDH*-mutant, patients, TTF was longer in cases with sub-median TBR_max_, whereas PRS did not differ significantly (Supplementary table 4). PRS was longer for patients with a sub-median TBR_max_ in the subgroup of *IDH*-mutant CNS WHO 2021 grade 2 and 3 gliomas, while TTF did not differ significantly. Similarly to median-split subgroups using SUV_max_, patients with a supra-median TBR_max_ had a higher CNS WHO 2021 grade, and larger FET TBR_max_, and contrast-enhancing volume (Supplementary table 5).

## Discussion

Glioma grading according to molecular features has improved prognostication in recent years, currently resulting in the 2021 revised edition of the WHO classification of CNS tumors [2]. Yet, prognosis for glioma patients remains poor, especially in the almost inevitable case of tumor recurrence. As no standard therapy for recurrent glioma is defined, treatment has to be tailored to the individual patient. For optimally fitting treatments, further markers of tumor aggressiveness are essential.

To optimally tailor treatments, [^18^F]FET PET has been established as a valuable imaging method to delineate tumor extent in vivo [8]. As a biomarker for prognostication uptake intensity on FET PET does not consistently predict survival [29], particularly not in glioblastoma [32]. In search of novel diagnostic and therapeutic tools, TSPO has gained interest recently, and earlier works could indeed show an association of tracer uptake on TSPO PET with *IDH* mutation status as a marker of glioma aggressiveness [24, 25]. To our knowledge, this is the first study analyzing the prognostic value of TSPO PET using [^18^F]GE180 in a larger cohort of recurrent glioma patients.

Here, we could confirm an association of [^18^F]GE180 uptake with known markers of malignancy such as histological tumor grade and, in the subgroup of IDH-wild-type glioblastoma, with the number of recurrences. Interestingly, as opposed to the primary situation [25], both recurrent *IDH*-mutant and *IDH*-wild-type glioblastoma show a highly increased maximum uptake value. This discrepancy between tumors in the primary and recurrent setting might have to do with an increased aggressiveness of recurrent IDH-mutant tumors as opposed to the primary situation (only 8 of 46 recurrent IDH-mutant tumors in our cohort did not show histological features of malignization).Whether hypermutation [33] or immune modulation induced by previous therapies drives a change in either TSPO expression or activation remains to be described in detail.

We found a strong negative correlation with survival time: Survival was more than three times longer in patients with sub-median SUV_max_ compared with those with supra-median SUV_max_. TTF was also significantly longer in cases with sub-median SUV_max_. Notably, this association between [^18^F]GE180 PET signal intensity and poor outcome was also found within the subgroups of *IDH*-wild-type and *IDH*-mutant tumor patients. A significant difference in TTF and PRS could even be seen in the largest homogenous patient subgroup of *IDH*-mutant astrocytoma CNS WHO 2021 grade 3, possibly due to a more nuanced substratification of malignancy than through the cut-off values set by histological grading. This clear association with survival even within molecularly homogenous subgroups suggests an *added* value of [^18^F]GE180 PET imaging to the clinically established molecular tumor stratification. This is notable because although molecular stratification greatly improved prognostication, diverging clinical courses are seen, especially after tumor recurrence, often but not always showing more malignant courses. If [^18^F]GE180 PET allowed further sub-stratification, therapy regimens and control intervals could be optimized.

Comparing these prognostically different groups of patients with low SUV_max_ versus high SUV_max_, significant differences were particularly found for the tumor size measured by contrast-enhanced MRI, and, among *IDH*-mutant glioma patients, also measured by [^18^F]FET PET-based tumor volume. Patients with high SUV_max_ had significantly larger contrast-enhancing tumor volumes, and it is tempting to speculate about a causal relationship between these parameters (e.g., high TSPO expression leads to fast tumor growth). Preclinical studies implicating a role of the TSPO protein in cellular functions such as reduced apoptosis [34], increase of proliferation [35], and cell migration [36] provide possible mechanistic explanations. However, only a low to moderate association could be found between [^18^F]GE180 SUV_max_ values and volume of contrast enhancement or [^18^F]FET PET-based tumor volume. Another conspicuity was the higher uptake intensity on [^18^F]FET PET in the group of patients with high [^18^F]GE180 SUV_max_, which can be explained by a moderate degree of correlation between both parameters. However, the strong association with survival outcomes was restricted to the [^18^F]GE180 PET signal and not found for uptake intensity on [^18^F]FET PET, which is in line with previous data demonstrating that TBR_max_ on [^18^F]FET does not serve as reliable prognostic biomarker [37, 38].

Despite promising survival data hinting at a role of TSPO in glioma tumorigenesis and progression [20, 39], the histological and molecular equivalent of a high TSPO tracer uptake remains to be evaluated in detail. Histologically and on mRNA level, tumor cells express high levels of TSPO, especially glioblastoma as opposed to low-grade glioma [19, 39]. This difference in TSPO expression even occurs among glioblastoma and other homogeneous molecular groups and correlates with higher tumor aggressiveness [20]. Although the mechanisms leading to this phenomenon are as yet unclear, an association with regulation of proliferation, apoptosis, migration, and/or mitochondrial functions such as respiration and oxidative stress regulation can be speculated [18]. While tracer uptake on [^18^F]FET PET is considered as surrogate marker of tumor cells due to overexpression of l-amino acid transporters particularly on tumor cells, upregulated TSPO expression in glioma is not only found in tumor cells but likewise in tumor-associated macrophages, endothelial cells, pericytes, and especially microglia [39]. As a microglia activation marker, TSPO PET visualizes neuroinflammation and has been established as a tool for imaging inflammatory CNS processes in neurodegenerative diseases [40, 41] or in multiple sclerosis [13, 42]. In the tumor microenvironment, inflammatory processes are increasingly recognized to play a role in gliomagenesis [43], treatment resistance [44], and tumor recurrence [12, 45]. As effectors of these processes, immunosuppressive myeloid-derived suppressor cells and regulatory T-cells outweigh activating immune effector cells such as T-cells and natural killer cells [46–49]. This mainly immunosuppressive glioma microenvironment is maintained and reinforced by expression and secretion of immune-suppressing molecules by glioma cells and tumor-associated astrocytes [10]. Nevertheless, the tumor microenvironment and immune status is highly heterogeneous across tumor entities: For example, immunosuppression more strongly prevails in *IDH*-wild-type glioblastoma, whereas *IDH*-mutant astrocytomas secrete granulocyte colony-stimulating factor which increases the ratio of non-suppressive neutrophils [11]. Yet, heterogeneity is not only seen between different molecular tumor entities, but also both spatially and temporally within the same tumor [50]. This heterogeneity adds to the difficulty of therapeutically stimulating an anti-glioma immune response [44]. Therefore, illustrating the tumor immune environment in vivo and monitoring changes over time is promising for prognostication and especially in light of recent advances in immunomodulating therapies [51].

A possible perspective would be to use [^18^F]GE180 PET imaging to augment standard FET PET imaging to guide treatment aggressiveness: Undertreatment is fatal in glioma recurrence, yet overtreatment is also to be avoided to minimize side effects and maintain quality of life in a situation where no standard treatment exists and treatment is mainly guided by clinical experience and individual, patient- and tumor-specific factors. In vivo information about tumor aggressiveness is therefore highly valuable for an informed treatment decision. Furthermore, non-invasive characterization of tumor heterogeneity could prompt multimodal treatment decisions such as resection or high-dose re-radiotherapy of especially aggressive but locally treatable areas. Scanning intervals could also be optimized, especially in histologically low-grade tumors showing signs of increased aggressiveness during initial or follow-up imaging.

Some limitations of this study must be noted. As it contains an unselected population of glioma patients undergoing molecular imaging, statistical power is limited by low numbers in individual subgroups. However, evaluating these homogeneous subgroups is extremely important to assess the *added* value of [^18^F]GE180 PET to routine neuropathological and molecular assessment. While further improvements in prognostication might be attained by extraction of radiomic or pharmacokinetic features, this study chose a straightforward method for image analysis, which is easily applicable in the clinical routine. As a purely observational study, the evaluated associations are not statistically influenced by therapy. As a prognostic value of [^18^F]GE180 PET could be shown in recurrent glioma patients, a longitudinal analysis of individual patients would be of high interest to address changes of TSPO expression and their prognostic value: An ideal study should obtain serial [^18^F]GE180 PET imaging of a cohort of, e.g., IDH wild-type glioblastoma patients prior to initial resection, before and after radiochemotherapy, during and after adjuvant chemotherapy, and at the time of (suspected) recurrence. It would be highly valuable to monitor changes in TSPO expression during disease progression and its association with individual survival to better understand tumor- as well as treatment- or patient-specific influences. These data would be boosted greatly by tissue samples at the individual timepoints to disentangle the cellular sources of the TSPO signal over time, particularly the proportions of tumor cells vs. inflammatory cells. Unlike in a human study, where tissue sampling is ethically warranted in case of newly diagnosed tumors and suspected recurrence only, an analogous animal study could include tissue sampling during and after therapy. This would be especially useful to determine how TSPO expression and its cellular distribution changes during treatment as well as early and later after treatment, and if an increase at different timepoints has to be interpreted differently, such as in the context of an inflammatory reaction to treatment [52, 53]. These aspects will be covered in upcoming studies.

## Conclusion

TSPO PET with [^18^F]GE180 is a promising imaging tool for prognostication in patients with recurrent *IDH*-mutant and *IDH*-wild-type glioma. The biological background and the usefulness of TSPO PET as a personalized read-out need to be elucidated in mechanistic and prospective studies.

## Supplementary Information

Below is the link to the electronic supplementary material.

Supplementary file1 (DOCX 217 KB)
