# Supplementary material for: TSPO PET signal using [18F]GE180 is associated with survival in recurrent gliomas
Source: Eur J Nucl Med Mol Imaging. 2022 Nov 4;50(3):859–69. doi: 10.1007/s00259-022-06006-1 (PMC9852133; doi:10.1007/s00259-022-06006-1)
Supplement: Supplementary file 1 — Supplementary file1 (DOCX 217 KB) [file 259_2022_6006_MOESM1_ESM.docx]

## **SUPPLEMENTAL DATA**

**Supplementary figure 1** Survival according to uptake intensity on [^18^F]FET PET (TBR_max_).


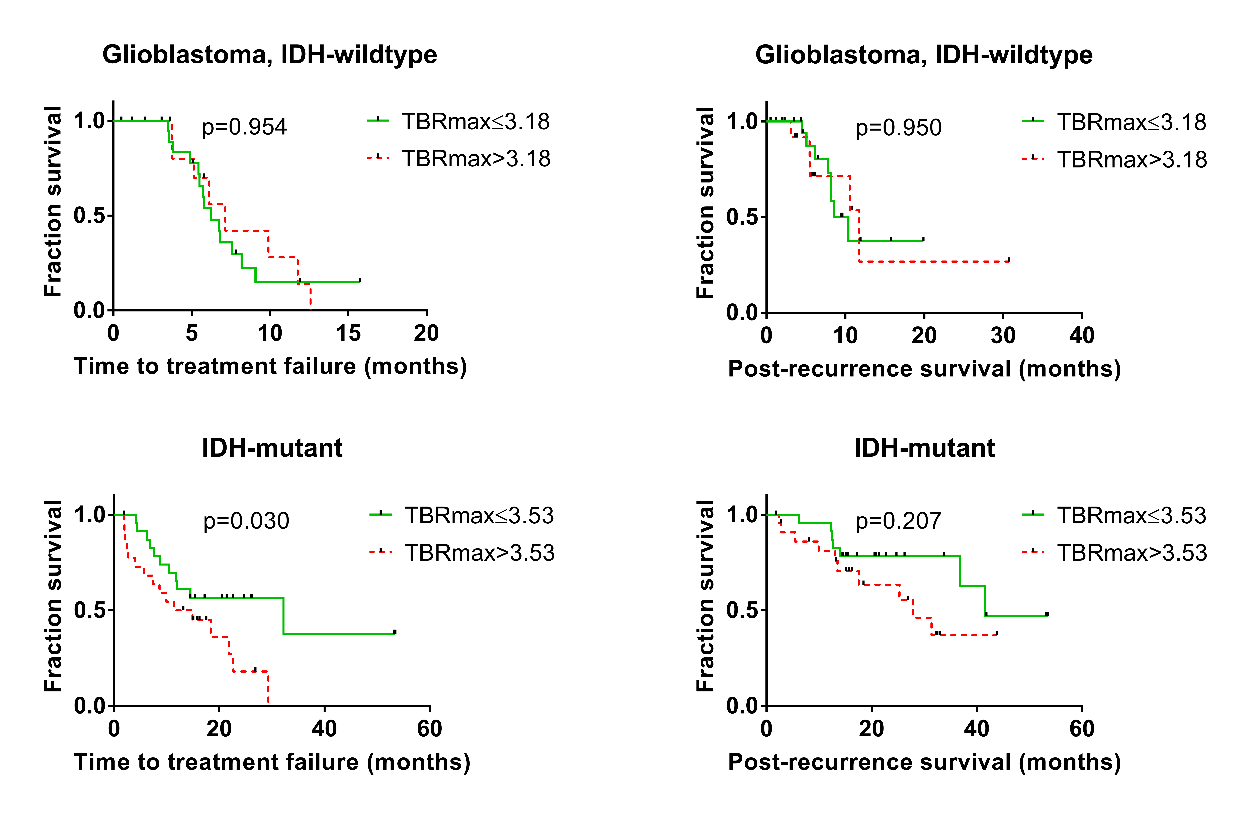


TBR_max_ – maximum tumor-to-brain-ratio, IDH – isocitrate dehydrogenase, TERT – telomerase reverse transcriptase

**Supplementary figure 2** Survival of CNS WHO 2021 Grade 4 glioma according to [^18^F]GE180 uptake

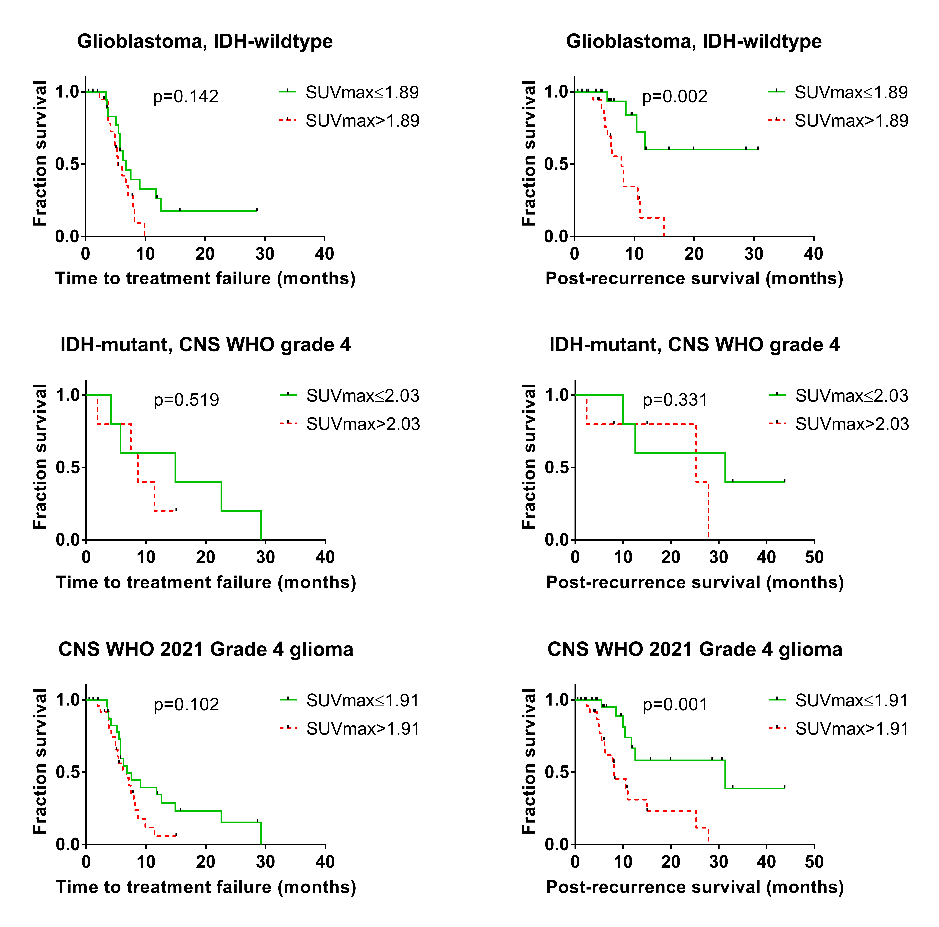


Supplementary table 1 [^18^F]GE180 PET SUV_max_ and survival outcome according to number of recurrence

| All recurrent glioma cases | SUV_max_ (median (range)) | P-value | PRS (median; months) | P-value | TTF (median; months) | P-value |
| --- | --- | --- | --- | --- | --- | --- |
| All diagnoses (n=88)  First recurrence (n=46)  Second recurrence (n=25)  Third or later recurrence (n=17) | 1.68 (0.59-4.36)  1.60 (0.59-3.34)  1.65 (0.61-3.82)  2.15 (0.72-4.36) | 0.159 | 27.9  31.4  36.9  14.0 | 0.532 | **8.7**  **11.8**  **9.9**  **5.3** | **0.001** |
| All IDH-wildtype (n=42)  First recurrence (n=28)  Second recurrence (n=11)  Third or later recurrence (n=3) | **1.89 (0.85-3.83)**  **1.67 (0.85-3.34)**  **2.01 (1.40-3.08)**  **2.82 (2.32-3.83)** | **0.024** | 10.6  10.6  8.2  15.0 | 0.504 | 6.1  5.2  6.1  5.3 | 0.291 |
| All IDH-mutant (46)  First recurrence (n=18)  Second recurrence (n=14)  Third or later recurrence (n=14) | 1.60 (0.59-4.36)  1.46 (0.59-2.76)  1.23 (0.61-3.82)  1.98 (0.72-4.36) | 0.296 | 36.9  41.6  36.9  13.5 | 0.055 | **18.4**  **29.3**  **Not reached**  **5.7** | **<0.001** |

SUV_max_ – maximum standardized uptake value, IDH – isocitrate dehydrogenase

Supplementary table 2 [18F]GE180 TBRmax in the examined patient population.

| All recurrent gliomas | TBR_max_  (median (range)) | P-value |
| --- | --- | --- |
| Overall (n=88) | 3.87 (1.45-13.63) |  |
| Male (n=56)  Female (n=32) | 4.35 (1.59-8.36)  3.28 (1.45-13.63) | 0.100 |
| CNS WHO 2021 grade 2 (n=8)  CNS WHO 2021 grade 3 (n=28)  CNS WHO 2021 grade 4 (n=52) | **2.15 (1.73-4.15)**  **3.16 (1.45-13.63)**  **4.44 (2.13-8.15)** | **0.022** |
| IDH-mutant (n=46)  IDH-wildtype (n=42) | 3.80 (1.45-13.63)  4.39 (2.13-8.15) | 0.291 |
| IDH mut. -1p/19 codel (n=28)  IDH mut. +1p/19 codel (n=18) | 4.06 (1.59-8.36)  2.75 (1.45-3.63) | 0.457 |
| IDH wt, TERT wildtype (n=5)  IDH wt, TERT mutant (n=22) | 3.20 (3.07-8.15)  4.44 (2.58-7.21) | 0.633 |
| IDH wt, MGMT methylated (n=19)  IDH wt, MGMT unmethylated (n=22) | 4.51 (2.13-8.15)  3.66 (2.13-8.15) | 0.132 |
| Low-affinity binding status (n=7)  Medium-affinity binding status (n=27)  High-affinity binding status (n=44) | 6.56 (1.78-7.73)  3.95 (1.59-7.00)  3.65 (1.45-8.36) | 0.105 |

*TBR_max_*  – *maximum tumor-to-background-ratio, CNS WHO 2021* – *World Health Organization Classification of Tumors of the Central Nervous System, IDH* – *isocitrate dehydrogenase, TERT* – *telomerase reverse transcriptase*

Supplementary table 3 Treatment regimens in patients with lower or higher than median [^18^F]GE180 TBR_max_

|  | TBR_max_ ≤ median (n; %) | TBR_max_ > median (n; %) | P-value |
| --- | --- | --- | --- |
| IDH-wildtype (n=42)  Radio-and chemotherapy  Radiotherapy only  Chemotherapy only  Experimental/others  No tumor-specific therapy | 21 (100)  6 (28.6)  8 (38.1)  4 (19.0)  0 (0.0)  3 (14.3) | 21 (100)  6 (28.6)  7 (33.3)  6 (28.6)  2 (9.5)  0 (0.0) | 0.243 |
| IDH-mutant (n=46)  Radio-and chemotherapy  Radiotherapy only  Chemotherapy only  Experimental/others | 24 (100)  8 (33.3)  12 (50.0)  4 (16.7)  0 (0.0) | 22 (100)  6 (27.3)  7 (31.8)  8 (36.4)  1 (4.3) | 0.278 |

TBR_max_  – maximum tumor-to-background-ratio, IDH – isocitrate dehydrogenase

Supplementary table 4 Survival of recurrent glioma patient groups according to [^18^F]GE180 TBR_max_.

| All recurrent glioma cases | PRS (median; months) | P-value | TTF (median; months) | P-value |
| --- | --- | --- | --- | --- |
| All diagnoses (n=88)  TBR_max_ ≤ 3.87 (n=44)  TBR_max_ > 3.87 (n=44) | 27.9  41.6  17.5 | 0.051 | 8.7  10.5  8.2 | 0.130 |
| All IDH-wildtype (n=42)  TBR_max_ ≤ 4.39 (n=21)  TBR_max_ > 4.39 (n=21) | 10.6  11.8  8.2 | 0.150 | 6.1  5.4  6.8 | 0.331 |
| All IDH-mutant (46)  TBR_max_ ≤ 3.80 (n=24)  TBR_max_ > 3.80 (n=22) | 36.9  41.6  27.9 | 0.158 | **18.4**  **32.2**  **11.4** | **0.039** |
| All astrocytoma, IDH-mutant (28)  TBR_max_ ≤ 4.06 (14)  TBR_max_ > 4.06 (14) | 27.9  36.9  27.9 | 0. 597 | **11.7**  **32.2**  **8.7** | **0.037** |
| Astrocytoma WHO 2021 grade 3, IDH mutant (17)  TBR_max_ ≤ 3.80 (9)  TBR_max_ > 3.80 (8) | **36.9**  **36.9**  **13.1** | **0.037** | 11.7  14.5  2.7 | 0.215 |
| All low-grade (2 or 3) astrocytoma, IDH-mutant (18)  TBR_max_ ≤ 3.36 (9)  TBR_max_ > 3.36 (9) | **36.9**  **36.9**  **13.1** | **0.003** | 14.5  32.2  6.2 | 0.063 |
| All oligodendroglioma, IDH-mutant and 1p/19q codeleted (18)  TBR_max_ ≤ 2.75 (9)  TBR_max_ > 2.75 (9) | Not reached  Not reached  Not reached | 0.464 | Not reached  Not reached  18.4 | 0.130 |

*TBR_max_*  – maximum tumor-to-background-ratio*, IDH – isocitrate dehydrogenase; PRS – post recurrence survival; TTF – time to treatment failure*

Supplementary table 5 Characteristics of high and low [^18^F]GE180 TBR_max_ groups.

|  | TBR_max_ ≤ median  (n; % or median; range) | TBR_max_ > median  (n; % or median; range) | P-value |
| --- | --- | --- | --- |
| IDH-wildtype (n=42)  Male / female sex  MGMT methylated / unmeth. (n=41)  TERT wildtype / mutant (n=27)  Age  [^18^F]FET TBR_max_ (n=35)  [^18^F]FET tumor volume ( n=35)  T2 volume  Contrast volume | 21 (50.0)  13 / 8 (31.0 / 19.0)  8 / 12 (19.5 / 29.3)  3 / 10 (11.1 / 37.0)  56.5 (32.3-70.0)  3.02 (1.55-3.79)  9.54 (0.0-76.66)  50.30 (0.0-198.10)  3.54 (.27-66.20) | 21 (50.0)  16 / 5 (38.1/69.0)  11 / 10 (26.8 / 24.4)  2 / 12 (7.4 / 44.4)  54.6 (30.8-70.2)  3.35 (2.47-5.28)  27.18 (7.68-124.75)  80.10 (24.20-337.3)  18.50 (0.0-85.6) | 0.317  0.427  0.557  0.560  **0.008**  0.074  0.054  **0.006** |
| IDH-mutant (n=46)  Male / female sex  CNS WHO 2021 grade 2 / 3 / 4  MGMT methylated / unmeth.  TERT wildtype / mutant (n=28)  Age  [^18^F]FET TBR_max_ (n=42)  [^18^F]FET tumor volume (n=42)  T2 volume  Contrast volume | 24 (52.2)  12 / 12(25.0 / 25.0)  7 / 16 / 1 (15.2 / 34.8 / 2.2)  21 / 3 (45.7 / 6.5)  9 / 6 (32.1 / 21.4)  47.6 (23.6-66.2)  2.51 (1.21-5.64)  5.14 (0.0-172.04)  51.30 (12.40-226.50)  0.07 (0.0-20.80) | 22 (47.8)  15 / 7 (32.6 / 15.2)  1 / 12 / 9 (2.2 / 26.1 / 19.6)  17 / 5 (37.0 / 10.9)  10 / 3 (35.7 / 10.7)  40.5 (29.1-71.9)  3.79 (2.88-7.49)  33.08 (1.67-120.69)  60.40 (9.13-253.90)  11.50 (0.0-61.2) | 0.211  **0.003**  0.361  0.339  0.511  **0.001**  **0.029**  0.499  **<0.001** |
| Astro, IDH mut., grade 3 (n=17)  Male / female sex  MGMT methylated / unmeth.  Age  [^18^F]FET TBR_max_  [^18^F]FET tumor volume  T2 volume  Contrast volume | 9  7 / 2 (41.2 / 11.8)  7 / 2 (41.2 / 11.8)  42.3 (29.9-56.3)  2.01 (1.56-5.64)  3.21 (0.00-172.04)  31.6 (12.40-226.50)  0.00 (0.00-6.09) | 8  6 / 2 (35.3 / 11.8)  5 / 3 (29.4 / 17.6)  37.7 (30.4-57.6)  3.79 (3.08-5.86)  71.96 (11.44-120.69)  63.50 (39.10-110.10)  18.55 (6.63-61.20) | 0.893  0.490  0.321  **0.019**  0.209  0.654  **0.005** |

TBR_max_  – maximum tumor-to-background-ratio, IDH – isocitrate dehydrogenase, TERT – telomerase reverse transcriptase, [^18^F]FET – [^18^F]Fluoroethyltyrosine, TBR_max_ – maximum tumor-to-brain-ratio, CNS WHO 2021-World Health Organization Classification of Tumors of the Central Nervous System
